# First-Order Derivative Spectrophotometry for Simultaneous Determination of Vitamin C and Nicotinamide: Application in Quantitative Analysis of Cocrystals

**DOI:** 10.1021/acsomega.4c03172

**Published:** 2024-06-20

**Authors:** Clóvis A. Balbinot Filho, Renata F. Teixeira, Jônatas L. Dias, Evertan A. Rebelatto, Marcelo Lanza

**Affiliations:** Department of Chemical and Food Engineering, Federal University of Santa Catarina, UFSC, PO Box 476, 88040-900 Florianópolis, SC, Brazil

## Abstract

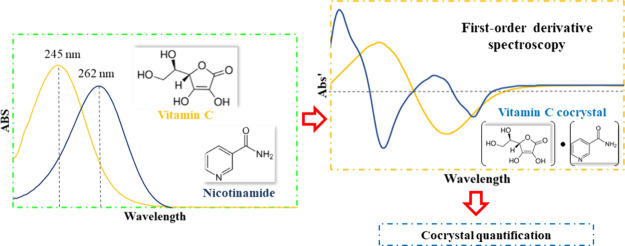

Vitamin C (l-ascorbic acid, ASC) and the amide form of vitamin B3 nicotinamide (NIC) can form cocrystals through hydrogen bonding. Currently, there is a lack of fast and reliable alternatives for precisely quantifying cocrystal components and their purity. Spectrophotometric analysis for quantifying such vitamin preparations is challenging due to overlapping absorbance bands in a narrow wavelength range in the ultraviolet (UV) region. Moreover, ASC undergoes progressive degradation if not diluted in a proper medium, requiring stability during quantitative analysis. This study adopted a fast, simple, and reliable two-component spectrophotometric assay for simultaneously determining ASC and NIC based on the first-order derivative spectrophotometry (FODS) method using sodium oxalate as a stabilizer for vitamin C. The FODS method showed linearity between 2 and 24 μg·mL^–1^ and good precision. The standard addition method was employed to validate FODS, with high recovery percentages (96.5 to 102.4% for ASC and 95.3 to 101.9% for NIC). The FODS method was successfully applied to quantify ASC and NIC in bulk powder produced by the gas antisolvent method. The proposed method could estimate cocrystal purity through mass balance regarding the expected 1:1 stoichiometry, confirmed by PXRD and DSC. Cocrystal purity determined by the FODS method (58–100%) aligned well with results from LC–MS (62–100%), with an accuracy exceeding 97%. The FODS method is as sensitive and accurate as high-performance liquid chromatography for simultaneously determining vitamin concentrations deriving from cocrystals. However, it is less costly, more efficient, and a suitable alternative to classical solid-state methods for estimating cocrystal purity.

## Introduction

Various analytical techniques have been developed for vitamin C (l-ascorbic acid, ASC) analysis,^1^ most of which are colorimetric methods based on quantification under visible light (400–700 nm). These methods rely on forming colored complexes of ASC with chromogenic reagents through redox reactions.^2,3^ Other analytical methodologies include classic titrimetry,^2,4^ chromatography,^5^ and electrochemical^6^ methods suitable for ASC determination in various matrices, including fruits, juice, and biological samples. Spectrophotometric methods are preferred over titrimetric ones because of their higher sensitivity.^2,4,7^ However, chromogenic reactants such as potassium permanganate may lack specificity to vitamin C and react with other substances present in the samples, such as reducing sugars, leading to an increase in absorbance and thus overestimating the actual ASC content.^2,7^ Such interferences represent a common limitation of spectrophotometric methods based on measuring absorbance reduction in the visible region.^8^

Direct UV spectrophotometry is a fast and simple method for determining vitamin C content.^2^ However, this method is unreliable in complex samples due to background absorption and interference from other substances in the UV region, particularly in pharmaceutical formulations. While most ingredients in these preparations (e.g., vitamin C tablets) do not interfere with ASC determination, large interferences in the UV light absorption were found in the presence of other vitamins, including vitamin B3 (nicotinamide, NIC).^9,10^ In this context, derivative spectrophotometry methods involve converting zero-order spectra to their first or higher derivative spectrum, resulting in significant changes in the shape of the spectrum, separation of overlapped signals,^11^ and elimination of broad bands resulting from turbidity and matrix interference.^12^ Additionally, sample variations are registered in terms of peak amplitude in the derivative curves.^13^ For example, first-order derivative spectroscopy in the near-infrared region was employed to quantify vitamin C in medicines and resolve the overlapping spectra of the ingredients.^14^ In contrast, third-order derivative spectroscopy was used to quantify vitamins A and E without prior separation.^15^

Resolving complex spectral overlaps of pharmaceutical binary mixtures without prior separation is a difficult task.^16^ Ratio manipulating spectrophotometric methods are examples of remarkable techniques in addressing this issue, wherein first-order derivatives of normalized ratio spectra have been utilized to solve the analysis of pharmaceutical binary combinations with severely overlapped spectra,^17,18^ particularly when there are significant concentration differences between the components.^17^ The relative simplicity of normalized spectra, which represent the absorptivity of components against wavelength rather than their corresponding absorbance,^18^ offers significant advantages in resolving spectral overlap and implies straightforward and moderate procedures compared to conventional spectrophotometric methods.^17,18^ Derivative ratio spectrophotometry has been proposed as a simultaneous method allowing both drugs to be determined using the same manipulation steps rather than separately manipulating each component.^17^

Derivative spectrophotometric methods enhance the specificity and selectivity of direct spectrophotometric analysis of drugs in the presence of excipients, degradation products, and impurities.^13^ These techniques are notably useful for quantifying two analytes with unresolved absorbance bands.^19^ As they offer background correction, they could enhance resolution by eliminating the errors associated with baseline shifts and linearity dependence.^20^ Derivative spectrophotometry has been used to determine ASC in drug ternary mixtures and effervescent tablets.^21−23^ However, the ASC quantification in cocrystals using these techniques remains relatively limited.

**Figure 1:**
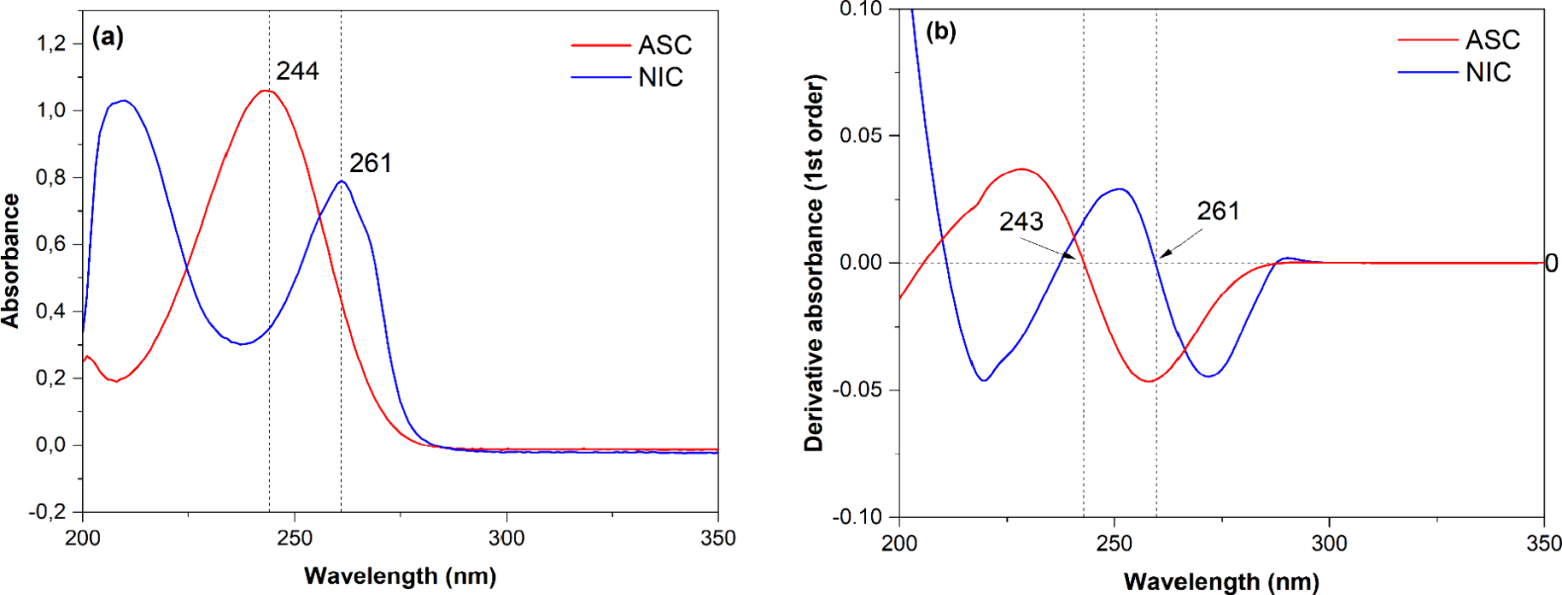
(a) Zero- and (b) first-order absorption spectra of pure vitamin C (ASC) and NIC 40 μg·mL^–1^ at pH 1.0.

Cocrystals are formed by two-component drug and/or bioactive complexes of molecules bound together by noncovalent interactions in the same crystalline lattice and at a defined stoichiometry.^24^ The ASC-NIC complex produces multivitaminic cocrystals at a 1:1 molar proportion.^25,26^ Accurate characterization and purity determination of pharmaceutical cocrystals are crucial to guarantee optimal performance.^27^ However, for routine quality control, the amount of both cocrystal constituents in a sample cannot be monitored by direct UV-spectrophotometry due to the overlapping absorbance peaks of ASC (243–245 nm) and NIC (261–262 nm),^9,28^ as shown in Figure 1a. Moreover, the instability of ASC when dissolved in water poses a major problem in its quantification,^9,29^ especially because it degrades more quickly in the presence of NIC,^10,28^ requiring methods to stabilize vitamin C against degradation for proper estimation. Stabilizers such as oxalic acid^9^ and alanine^29^ prevent ASC’s complete hydrolysis to final degradation products and its underestimation in spectrophotometric assays.

Various studies^14,30,31^ employed a two-component spectrophotometric method to conveniently determine ASC in solution and the presence of interferents by FODS. Regarding cocrystals, Biscaia et al.^19^ successfully validated the FODS method for quantifying cocrystal components lamotrigine and NIC in the UV region, and Neurohr et al.^32^ proposed a method for determination of cocrystal purity based on a mass balance and the quantification of the individual parent components by HPLC. This method was further validated by Ercicek et al.^33^ and showed reliable results compatible with established methods (i.e., quantitative phase analysis by X-ray diffraction).

In this context, the main objective of this work is to develop and validate the FODS method for the quantification of cocrystal parent compounds (ASC and NIC) by UV-spectrophotometry and apply this method to quantify the purity of ASC-NIC cocrystal samples by the mass balance proposed by Neurohr et al.,^32^ to provide a suitable, simple, reproducible and accurate protocol for cocrystal quantification.

## Materials and Methods

### Chemicals

Pure ASC (99.7% mass fraction, Neon Comercial) and NIC (99.0% mass fraction, Sigma-Aldrich) were obtained from suppliers without purification. A sodium oxalate solution (0.075% m/v) was prepared by dissolving 0.75 g oxalic acid in 1000 mL of a 0.1 M phosphate (KH_2_PO_4_–Na_2_HPO_4_) buffer, following previous studies.^9,29^ The pH was adjusted to 3.0 using a hydrochloric acid solution (0.1 N) measured by a calibrated benchtop pH meter (Hanna Instruments, USA) to prevent ASC oxidation to dehydroascorbic in solutions with a pH > 5.0.^34^ Sodium oxalate was the sole solvent utilized to dilute the samples. All components of the buffer solutions were of analytical grade, as were the carbon dioxide (CO_2_) and ethanol employed in the cocrystal synthesis.

### Cocrystal Sample Preparation

ASC-NIC cocrystals at a 1:1 stoichiometry were obtained in our ongoing study (unpublished data) through the gas antisolvent (GAS) method employing supercritical CO_2_ as described in detail elsewhere.^35,36^ Initially, ASC/NIC solutions were prepared by simultaneous dissolution of appropriate amounts of ASC and NIC in 30 mL of ethanol to produce solutions of 1:1, 1:2, and 2:1 ASC/NIC molar ratios. The solutions were filtered using a hydrophilic-PTFE syringe filter (0.45 μm) to remove dust. The amount of parent components used to form the solutions were as follows: to obtain a 1:1 ASC/NIC molar ratio solution, 176.12 mg of ASC and 122.12 mg of NIC were used; to obtain a 1:2 ASC/NIC molar ratio solution, 176.12 mg of ASC and 244.24 mg of NIC were used; and to obtain a 2:1 ASC/NIC molar ratio solution, 352.24 mg of ASC and 122.12 mg of NIC were used.

Each ASC/NIC solution was subsequently placed into a stainless-steel jacketed vessel and pressurized by pumping CO_2_ (20 MPa, 3–5 °C) with the outlet valves closed until the processing pressure and temperature were reached (up to 9 MPa at 45 °C^37^). After 10 min of mild stirring, the outlet valve was opened, and approximately 1 L of CO_2_ was continuously pumped at a flow rate of 10 mL min^–1^ to dry out the ethanol and precipitate fine particles, resulting in a solvent-free cocrystal bulk powder.

### Cocrystal Characterization

#### Powder X-ray Diffraction (PXRD)

Crystalline identification of the material was acquired from a benchtop powder diffractometer (MiniFlex600, Rigaku, USA), operating with a copper radiation source (40 kV voltage and 15 mA current). Measurements were taken at room temperature by angular scanning in the θ–2θ mode between 2° and 50° with a step size of 0.02° 2θ and scanning speed of 10°/min.

#### Differential Scanning Microscopy (DSC)

Thermal analysis was performed in a Jade differential calorimeter scanner (PerkinElmer, USA). Samples (∼5 mg) were placed in sealed aluminum pans and heated (40–300 °C) under a nitrogen atmosphere (60 mL/min flow), and scans were run in a single heating cycle (no cooling cycle) at 10 °C/min. Thermal events were recorded as sharp endothermic peaks or shifts in the baseline.

### First-Order Derivative Spectroscopy (FODS) Method

#### Zero-Crossing Points

Following the methodology described by Biscaia et al.,^19^ a sweep scan was performed in the UV region from 200 to 400 nm (1 nm resolution) for each pure component (ASC or NIC) using 1 cm path-length quartz cuvettes. The recorded spectra were individually derived from zero- (Figure 1a) to the first-order (Figure 1b), relative to the absorbance, using the built-in mathematical operation “differentiate” in the UVWin 6 software (PG Instruments, UK). The first-order spectra of pure ASC and NIC were overlapped to find a wavelength at which each component shows zero derivative absorbance^19^ (Abs′ = 0), being 261 nm for ASC (Abs′_NIC_ = 0), and 243 nm for the quantification of NIC (Abs′_ASC_ = 0) at acidic conditions (pH < 5.0) as depicted in Figure 1b.

#### Standard Calibration Curve

**Figure 2:**
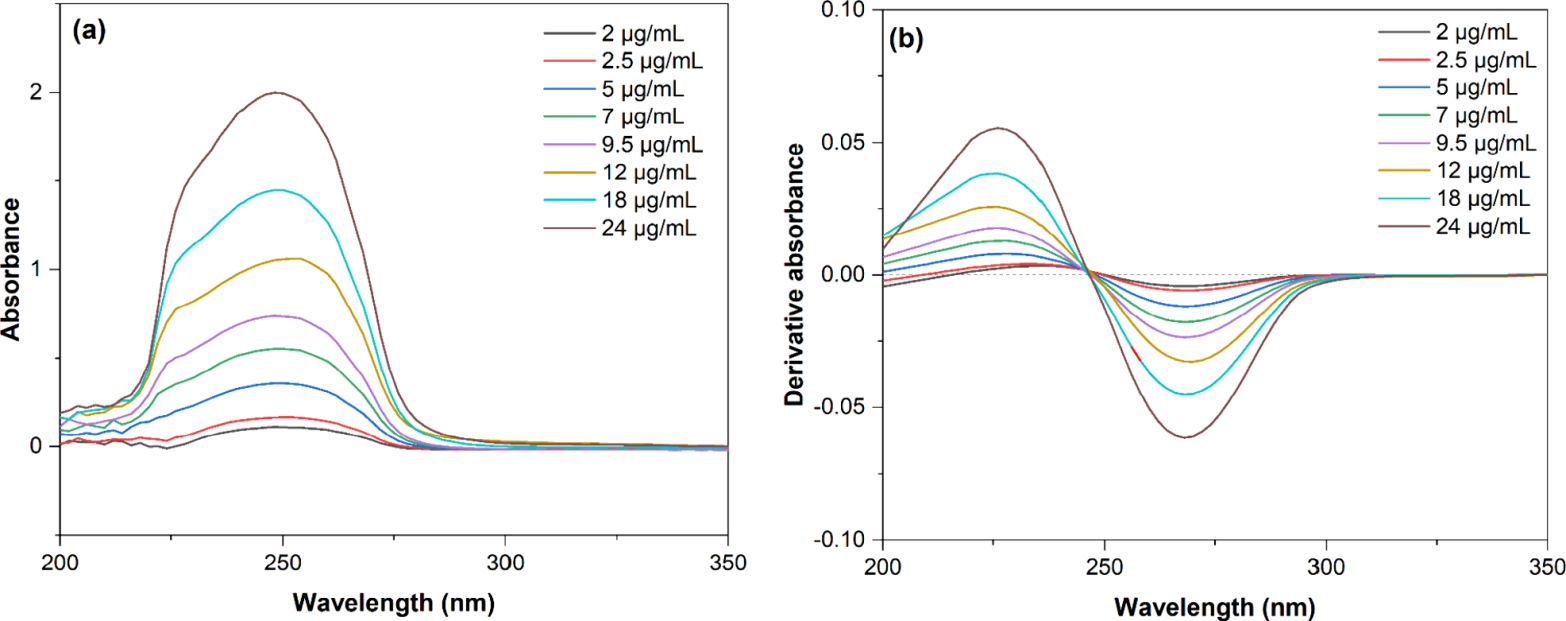
(a) Zero- and (b) first-order derivative absorption spectra of the calibration curves (2–24 μg·mL^–1^) of ASC and NIC.

Stock mixed solutions of ASC and NIC were prepared by accurately dissolving 100 mg of each solute in 250 mL of the sodium oxalate to a final concentration of 0.4 mg·mL^–1^. Serial standard dilutions ranging from 2 to 24 μg·mL^–1^ were then prepared from the stock solution by diluting in sodium oxalate and scanned from 200 to 400 nm. The resulting absorbance spectra of the mixture at each concentration are plotted in Figure 2a. The corresponding first derivative is presented in Figure 2b, and the derivative absorbance of each analyte in the sample (at 243 nm for NIC and 261 nm for ASC) is recorded. An independent calibration curve for each analyte is obtained and presented in the Supporting Information (Figure S1).

### Standard Addition Method and Precision

The ability of the FODS method to precisely quantify ASC and NIC in mixtures was investigated by mixing certain volumes of individual ASC and NIC solutions (0.4 mg·mL^–1^), dissolved to a final volume of 2 mL in sodium oxalate (Table 1). The standard addition method was also employed to assess the accuracy of the FODS method in recovering a standard amount added to mixes. Fresh standard solutions were prepared the next day by weighing 40 mg of each standard and dissolving in 100 mL sodium oxalate (≈0.41 mg·mL^–1^). Standard aliquots (2–7 μg·mL^–1^) were then added to the previous mix of the two analytes at a concentration of ≈4.8 μg·mL^–1^ each in sodium oxalate to reach concentrations within the linearity range of the method. Recovery percentages of each added standard given by the FODS method were recorded (Table 2). The method’s reproducibility was assessed by analyzing different mixtures of ASC and NIC at different concentrations (intraday) and the same concentrations prepared on two different days (interday), with precision expressed in terms of the variation of the analytical response.^7,19^ The limits of detection (LOD) and quantification (LOQ) of the method were calculated based on the slope of calibration curves (*a*) and the respective standard error (SD_a_), according to eqs 1 and 2.^38^
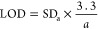
1
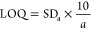
2

**Table 1:** Validation of the FODS Method Using Simple Mixtures of ASC and NIC

	ASC^a^				NIC^a^			
mix	vol. (μL)	concentration (μg·mL^–1^)		rec. (%)	vol. (μL)	concentration (μg·mL^–1^)		rec. (%)
		theoric	found			theoric	found	
1	200	4.80	4.88	101.8	200	4.75	4.67	98.3
2	200	4.80	4.93	102.7	100	2.38	2.35	98.7
3	400	9.61	9.57	99.7	100	2.38	2.39	100.4
4	300	7.21	7.16	99.4	400	9.51	9.45	99.4
5	300	7.21	7.25	100.7	600	12.96	12.69	97.9

a Mixes were prepared to a final volume of 2 mL in sodium oxalate from 48.04 μg·mL^–1^ ASC and 47.50 μg·mL^–1^ NIC solutions.

**Table 2:** Standard Addition Method for Recovering Added ASC and NIC to a Mixed Solution of Both Compounds Using the FODS Method

	ASC concentration (μg·mL^–1^)				NIC concentration (μg·mL^–1^)			
mix^a^	add	total	found	rec. (%)	add	total	found	rec. (%)
**1**	7.3	12.08	12.11	100.4	2.4	7.15	7.18	101.1
**2**	4.9	9.65	9.47	96.3	2.4	7.15	7.20	102.0
**3**	2.4	7.23	7.30	103.0	2.4	7.15	7.24	103.0
**4**	2.4	7.23	7.34	104.6	4.8	9.6	9.43	97.4
**5**	2.4	7.22	7.25	100.9	7.2	12.0	11.88	99.0

a To the previously prepared ASC and NIC mix (4.8 μg·mL^–1^), freshly prepared individual standards were added to a final volume of 2 mL of sodium oxalate.

### Cocrystal Sampling and Quantification

About 10 mg of the ASC–NIC cocrystals was diluted in 10 mL of sodium oxalate and then diluted again to fit the calibration curve. After sample scanning, the resulting first-order derivative spectra provide two values for the derivative absorbance at wavelengths corresponding to zero-crossing points for ASC and NIC. From the resulting ASC and NIC contents determined by the FODS method, a mass balance in each cocrystal batch was employed, following the work of Neurohr et al.^32^ to estimate the purity of the bulk obtained by GAS, considering the following assumptions:^32,39^

**Figure 3:**
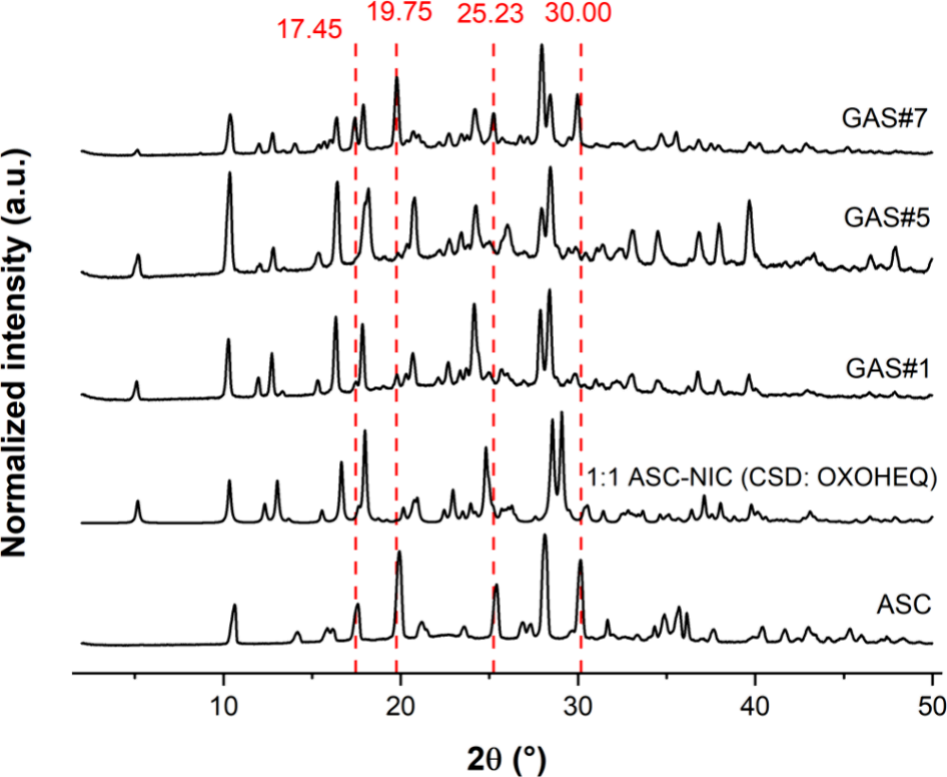
Powder X-ray diffraction patterns for cocrystal samples of different purities the simulated CCDC standard (CSD entry OXOHEQ) and pure ASC.

**Figure 4:**
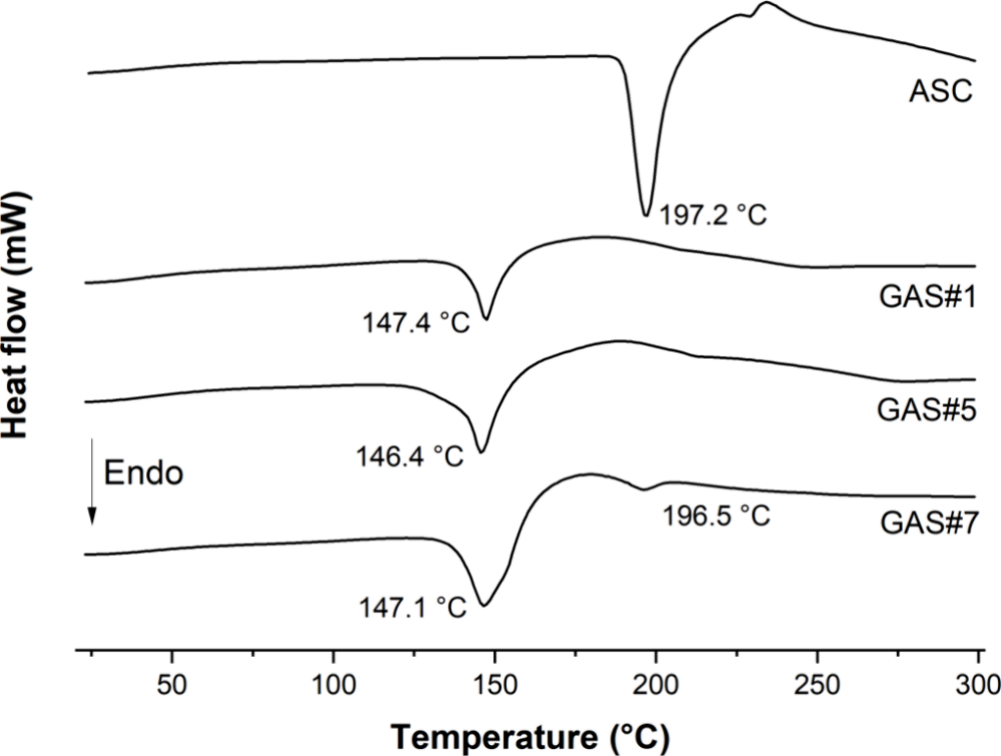
Differential calorimetry scanning for cocrystal samples and pure ASC showing thermal events at different temperatures.

1. The cocrystal stoichiometry of 1:1 (ASC:NIC) is fixed,^25^ as confirmed by PXRD and DSC (Figures 3 and 4);
2. Only ASC in excess to the cocrystal precipitates as homocrystals, while all NIC precipitates as a cocrystal according to by PXRD and DSC (Figures 3 and 4).

Therefore, cocrystal purity (wt %) can be obtained according to eq 3:^39^
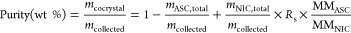
3

*m*_collected_ is the total mass recovered after the GAS process, *m*_ASC,_ and *m*_NIC,_ calculated from FODS/HPLC results. *R*_s_ is the expected stoichiometric ratio of the cocrystal (1.0), and MM is the molecular mass. Appendix A of the Supporting Information provides the detailed mass balance equations involved. Subsequently, results obtained by the FODS method were compared to HPLC to check the closeness in cocrystal purity calculated by the two methods.

### Liquid Chromatography Coupled with Mass Spectrometry (LC-MS)

The cocrystal samples utilized in the FODS method assessment were also analyzed by liquid chromatography coupled with mass spectrometry (LC-MS) using a mono quadrupole detector with dual ionization source (LCMS-2050 Model, Shimadzu, Japan) and LabSolutions software for data acquisition. A Shim-pack XR-ODS III column (150 × 2 mm) was employed as the stationary phase. Samples were dissolved (20 mg·L^–1^) in methanol HPLC-grade. Water (A) and acetonitrile (B), both with 0.1% (v/v) formic acid, were used as the mobile phase.^40^ The following eluent gradient was set: 10% A (0–2 min), 90–10% A (2–4 min), 10–90% A (4–5 min), and 10% A (5–10 min). Injections (1 μL) were performed at 0.3 mL·min^–1^ at 35 °C. The detection and quantification followed procedures described in the literature for LC-MS of water-soluble vitamins.^41,42^

MS detection operation conditions used in the interface were as follows: desolvation temperature (450 °C); nebulizing gas flow (2 L·min^–1^), drying gas flow (5 L·min^–1^), and heating gas flow (7 L·min^–1^). ASC- (anionic) and NIC- (cationic) derived ions were detected at different ionization modes. The obtained mass-to-charge ratio (*m*/*z*) fragments spectra at each retention time generated total ion chromatograms (TIC, Figure S2 of the Supporting Information) whose peak area was used to trace standard calibration curves (*R*^2^ > 0.999). An individual calibration curve for each standard component in the mixture (2–20 mg·L^–1^) was plotted, and concentrations of ASC and NIC were converted to the molar ratio. The quantified proportion between each cocrystal component by HPLC was used to compare the accuracy of the FODS method.

## Results and Discussion

### Methodology Development and Fitting

The absorbance (zero-order) spectrum of the serial dilutions for the ASC and NIC mixture (Figure 2a) at pH 3.1 exhibits a broad and intense peak centered at 250 nm, likely resulting from the overlap of ASC and NIC peaks maxima. The criterion for selecting optimal derivative order is to achieve enough separation of overlapped signals. Low derivative orders are expected to yield wide spectrum bands, while higher orders are suitable for narrow spectral bands.^11^ Despite a second-order derivative of absorbance providing a good resolution of ASC and NIC spectra (Supporting Information, Figure S3a), trials with higher-order derivative spectra were found not suitable for the quantification of these vitamins due to very straight zero-crossing points and very small values for the respective derivative absorbances, especially in the third-order derivative (Figure S3b). Therefore, first-order derivatization (Figure 1) was selected for the derivative spectrophotometric method.

Zero-crossing points for the FODS method were then defined at wavelengths where the first-order derivative absorbance of ASC crosses the zero line, and there is no influence of NIC and vice versa.^11,19^ The calibration curve is descending at 261 nm (Abs′_NIC_ = 0), and derivative absorbance values for ASC are all negative (Figure S1a), while at 243 nm (Abs′_ASC_ = 0), the calibration curve is ascending, showing that derivative absorbance values for NIC are all positive (Figure S1b), corroborating the curves of Figure 2b. The opposite trend is observed when analyzing curves obtained at pH > 5.0 (data not shown) since ASC oxidizes to DHA at pHs between 4.3 and 11.8. In contrast, the fully protonated vitamin C (ASC) form prevails at lower pHs.^34^ Therefore, the FODS method was validated at pH 3.0 to consider only the reduced and most biologically abundant form of vitamin C (ASC).

The calibration curves built on these points showed good linearity (*R*^2^ > 0.997), as presented in Figure S1. Analysis of variance (ANOVA, α = 0.05) showed that the linear regression was highly significant (*p* < 0.001), as evidenced by the high *F*-values and the low *p-*values (Table S1). The LOD and LOQ values represent the method’s sensitivity and outline a suitable range of concentrations to be determined.^19^ The low LOD for ASC (0.036 μg·mL^–1^) and NIC (0.090 μg·mL^–1^) and LOQ values of 0.108 and 0.37 μg·mL^–1^ found for ASC and NIC, respectively, indicate a high sensitivity of the method.

### Recovery Tests and Validation

Solutions of known concentrations of both compounds were diluted in sodium oxalate, and the concentrations of ASC and NIC determined by the FODS method are presented in Tables 1 and 2, expressed as % recovery of each analyte. The % recovery varied from 96.5 to 102.4% for ASC and 95.3 to 101.9% for NIC. The next day, new solutions of the same concentrations (≈0.4 mg·mL^–1^) of ASC and NIC were individually prepared, and known aliquots of each were added to the previous mixture (Table 2). Recovery percentuals agree with AOAC’s standardized guidelines for method performance assessment, considering the accepted variation (95–105%) relative to the analyte concentration range.^43^ Reproducibility values (*n* = 5) for intraday samples of varying concentrations, expressed in terms of the standard deviation (SD), were 1.37% (average recovery of 100.84%) for ASC and 0.98% (average recovery of 98.95%) for NIC. When considering interday samples of the same concentration, the variation for ASC (7.2 μg·mL^–1^, *n* = 3) was 0.79% (average recovery of 99.4%), while for NIC (2.4 μg·mL^–1^, *n* = 5), it was higher at 2.37% (average recovery of 99.8%). Therefore, the method was more accurate in determining the ASC content than NIC in mixed samples.

### Cocrystal Component Identification and Quantification

The FODS method, as well as HPLC, were employed to (i) quantify cocrystal components (ASC and NIC) from eight distinct GAS semibatches and (ii) estimate the bulk purity regarding the defined stoichiometric ratio of 1:1 for this cocrystal, as previously determined.^25,26^ According to Table 3, the FODS method and HPLC (LC-MS) agreed well regarding cocrystal purity (58–101%). GAS cocrystals presented high purity, except for run #7, which started with a solution at 2:1 ASC:NIC molar proportion and generated more impurity (ASC homocrystals) in the course of ASC exceeding the stoichiometric ratio. This hypothesis is supported by directly dividing ASC and NIC molar concentration by either HPLC or derivative spectroscopy using molar masses of ASC (176.12 g·mol^–1^) and NIC (122.2 g·mol^–1^), in which a value higher than 1.00 is likely to be obtained, indicating ASC impurities. Similarly, Biscaia et al.^19^ confirmed the 1:1 stoichiometry of lamotrigine-nicotinamide cocrystals of different batches based on the quantification of cocrystal samples by the FODS method. However, the authors did not make any inferences about the purity of the cocrystals.

**Table 3:** Application of the FODS Method to the Quantification of ASC and NIC and Cocrystal Purity^b^

cocrystal batch (collected mass, mg)	LC-MS^a^				FODS^a^			
	ASC_total_ (mg)	NIC_total_ (mg)	cocrystal mass (mg)	cocrystal purity (wt %)	ASC_total_ (mg)	NIC_total_ (mg)	cocrystal mass (mg)	cocrystal purity (wt %)
GAS#1 (220.9)	133.2	88.0	214.6	97.13	133.4	87.1	213.1	96.46
GAS#4 (153.5)	92.2	61.6	150.2	97.85	94.0	59.7	145.6	94.85
GAS#5 (196.4)	116.2	80.5	196.3	99.96	116.5	79.8	195.0	99.30
GAS#7 (342.5)	255.6	87.7	213.3	62.28	259.9	81.7	200.4	58.5
GAS#9 (194.2)	114.2	80.0	195.4	100.60	114.5	79.7	194.6	100.20
GAS#10 (239.3)	142.1	97.9	238.4	99.62	141.9	97.3	237.6	99.30
GAS#13 (134.6)	79.6	55.3	134.7	100.10	79.4	54.4	133.7	99.31
GAS#15 (213.2)	125.7	87.7	214.0	100.34	128.3	85.8	208.7	97.87

a Values represent a mean of two determinations.

b Purity regarding the 1:1 (ASC:NIC) cocrystal stoichiometry (confirmed by PXRD and DSC, Figures 3 and 4).

In addition, the novel molecular arrangement of ASC within the crystalline lattice as a cocrystal is evidenced by the new diffraction peaks at specific angles distinct from those of the pure (homocrystal) molecule^44^ (depicted as red lines in Figure 3). The patterns of cocrystals GAS#1 and GAS#5 closely align with the simulated standard deposited in the Cambridge Structure Database (CSD)^45^ compared to the less pure GAS#7 sample that exhibits either cocrystal or pure ASC peaks. Similarly, the DSC thermograms depicted in Figure 4 support the findings of Table 3. Cocrystal-pure samples display a unique thermal event as a sharp endothermic peak characteristic of cocrystals, occurring at an intermediary melting temperature between those corresponding to ASC and NIC (c.a. 146.5 °C).^46^ In contrast, GAS#7 manifests an additional event at the melting point of ASC (c.a. 196 °C), suggesting the presence of ASC impurities.

Interestingly, high-purity 1:1 cocrystals from runs #5, #9, and # 10 were produced from an initial 1:2 (ASC:NIC) molar ratio, indicating that contrary to ASC, excess NIC was solubilized and vented out, as previously reported in the literature for cocrystallization by GAS employing NIC as a coformer.^36,39^ PXRD and DSC results qualitatively corroborate the cocrystal quantification achieved through the FODS method and offer a theoretical basis to validate the developed methodology. Based on the quantitative and qualitative results, it is possible to confirm that the assumptions that all NIC is converted into cocrystals and that excess ASC remains as homocrystals in the bulk powder are correct. Moreover, from the results of Table 3, one can select GAS conditions that lead to purer cocrystals using the FODS method.

Nonetheless, the method adopted in this work presents an advancement over classical solid-state quantification of cocrystal components and purity,^47^ such as quantitative phase analysis (QPA PXRD). QPA faces significant challenges related to overlapping peaks in the diffractograms and polymorphism, especially in complex systems such as cocrystals that might include at least three species (the cocrystal and its parent compounds impurities), making it difficult to identify and quantify the present phases accurately. Sample preparation and instrumental operation parameters are decisive for obtaining good-quality PXRD data.^27,48^ Besides, the need for high-resolution diffractograms with well-defined peaks of high intensity to perform Rietveld diffractogram refinement^49^ implies a very time-consuming scan involving complex models to fit the data.

On the other hand, HPLC is considered a reference technique for solute quantification. However, it is a demanding analysis because of the more complex sample preparation and the need for specialized equipment and gradient solvents, which are more time-consuming and not environmentally friendly.^19^ In contrast, spectrophotometric methods are simpler, more accurate, and more cost-effective than chromatography to analyze mixtures containing two or more components without previous separation.^20^ Therefore, the FODS method provides a direct, comparable method to chromatography that does not require reaction time, and dilution in a proper solvent is the only procedure needed.

Moreover, methods based on the first-order derivative of ratio spectra performed comparably to chemometric methods involving multivariate calibration in recovering drugs from mixtures in pharmaceutical dosage formulations^20,21^ even in the presence of their degradation products.^20^ Spectrophotometric methods were simple, accurate, and precise regarding spectral resolution. Moreover, this enhanced sensitivity widens the opportunities for them to be used as stability-indicating methods in quality control.^16,20^ The FODS method proved suitable for cocrystal quantification, but not limited to, since it can aid in other types of analysis that require quantification of vitamin C, such as currently done for photostability assays of ASC in creams and gels using UV irradiation sources.^10,50^

## Conclusions

Due to a strict absorbance region, the analytical determination of vitamin preparations is impossible by direct UV–visible spectroscopy. The well-established first-order FODS method could be successfully adapted for the mutual quantification of pure vitamin C (ASC) and NIC or their cocrystalline form using aqueous sodium oxalate as a stabilizer/solvent. This relatively simple and sensitive method dismisses nocive reagents, separation, or separation steps. In addition, the mass of produced cocrystal and its purity in bulk could be conveniently estimated from the total contents of ASC and NIC in the batches provided by FODS through mass balance. The results are comparable to HPLC determinations and present a reliable way to perform an assay for simultaneous quantification of cocrystal components without requiring complex processes, also representing a suitable alternative to solid-state methods for cocrystal analysis in supramolecular chemistry.

## Supporting Information Available

Additional information to the text includes individual solute calibration curves, statistics for linear regressions, HPLC-MS validation parameters and corresponding chromatograms, higher-order derivative spectra of ASC and NIC, and equations for the mass balance and cocrystal purity determination (PDF)
