# Supplementary material for: First-Order Derivative Spectrophotometry for Simultaneous Determination of Vitamin C and Nicotinamide: Application in Quantitative Analysis of Cocrystals
Source: ACS Omega. 2024 Jun 20;9(26):28776–83. doi: 10.1021/acsomega.4c03172 (PMC11223235; doi:10.1021/acsomega.4c03172)
Supplement: Supplementary file 1 — ao4c03172_si_001.pdf [file ao4c03172_si_001.pdf]

Supporting Information

First-order derivative spectrophotometry for simultaneous determination of vitamin C and nicotinamide: Application in quantitative analysis of cocrystals

Clóvis A. Balbinot Filho*, Renata F. Teixeira, Jônatas L. Dias, Evertan A. Rebelatto, Marcelo Lanza

Department of Chemical and Food Engineering, Federal University of Santa Catarina, UFSC, PO Box 476, 88040-900, Florianópolis, SC, Brazil

clovis.filho@posgrad.ufsc.br *corresponding author

Table S1. ANOVA table for the linear regression[1].

Curve	Source of variation	DF	SS	*MS*	F value	*p-value*
ASC	*Model*	1	0.00222	0.00222	8,532.93	$4.59 \cdot 10^{-12}$
	Error	7	$1.83 \cdot 10^{-6}$	$2.61 \cdot 10^{-7}$		
	Total	8	0.00223			
NIC	*Model*	1	$8.55 \cdot 10^{-5}$	$8.55 \cdot 10^{-5}$	1,423.65	$2.95 \cdot 10^{-6}$
	Error	7	$2.40 \cdot 10^{-7}$	$6.01 \cdot 10^{-8}$		
	Total	8	$8.58 \cdot 10^{-5}$			

[1]DF = Degrees of freedom, SS = Sum of squares, MS = Mean square.

Table S2. Validation data for vitamin C and nicotinamide determination by HPLC.

	**Ascorbic acid**	**Nicotinamide**
Retention time (min) ± SD	1.097 ± 0.003	1.076 ± 0.004
Linearity range ($mg \cdot L^{-1}$)	7-20	2-18
Correlation coefficient (R^2)	0.999	0.997
Slope	48,940	365,412
Intercept	-184,420	-98,847
Precision (%)	99.68 ± 6.07	100.36 ± 5.24
LOD ($mg \cdot L^{-1}$)	0.15-0.29	0.02-0.04
LOQ ($mg \cdot L^{-1}$)	0.45-0.61	0.05-0.13

[1]Values represent a mean of five determinations. SD standard deviation.

Figure S1. Standard calibration (analyte concentration vs. Abs') curves obtained for **(a)** vitamin C (ASC) at 261 nm and (**b)** nicotinamide (NIC) at 243 nm from mixed solutions at pH 1.0.

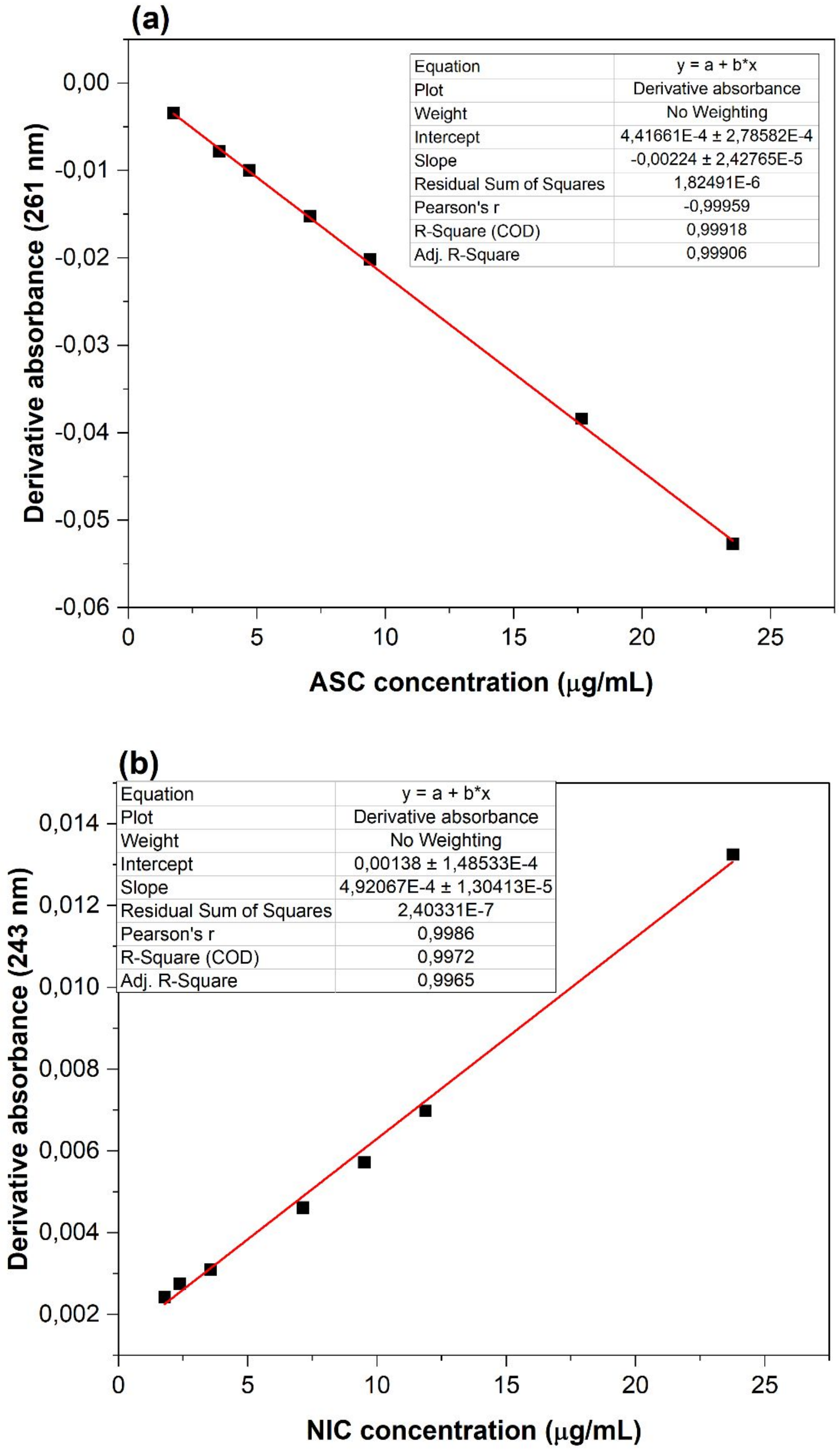

Figure S2. HPLC chromatograms and the respective fragmentation patterns for **(a)** ascorbic acid (vitamin C) and **(b)** nicotinamide detected from LC-MS at distinct ionization modes.

(a)

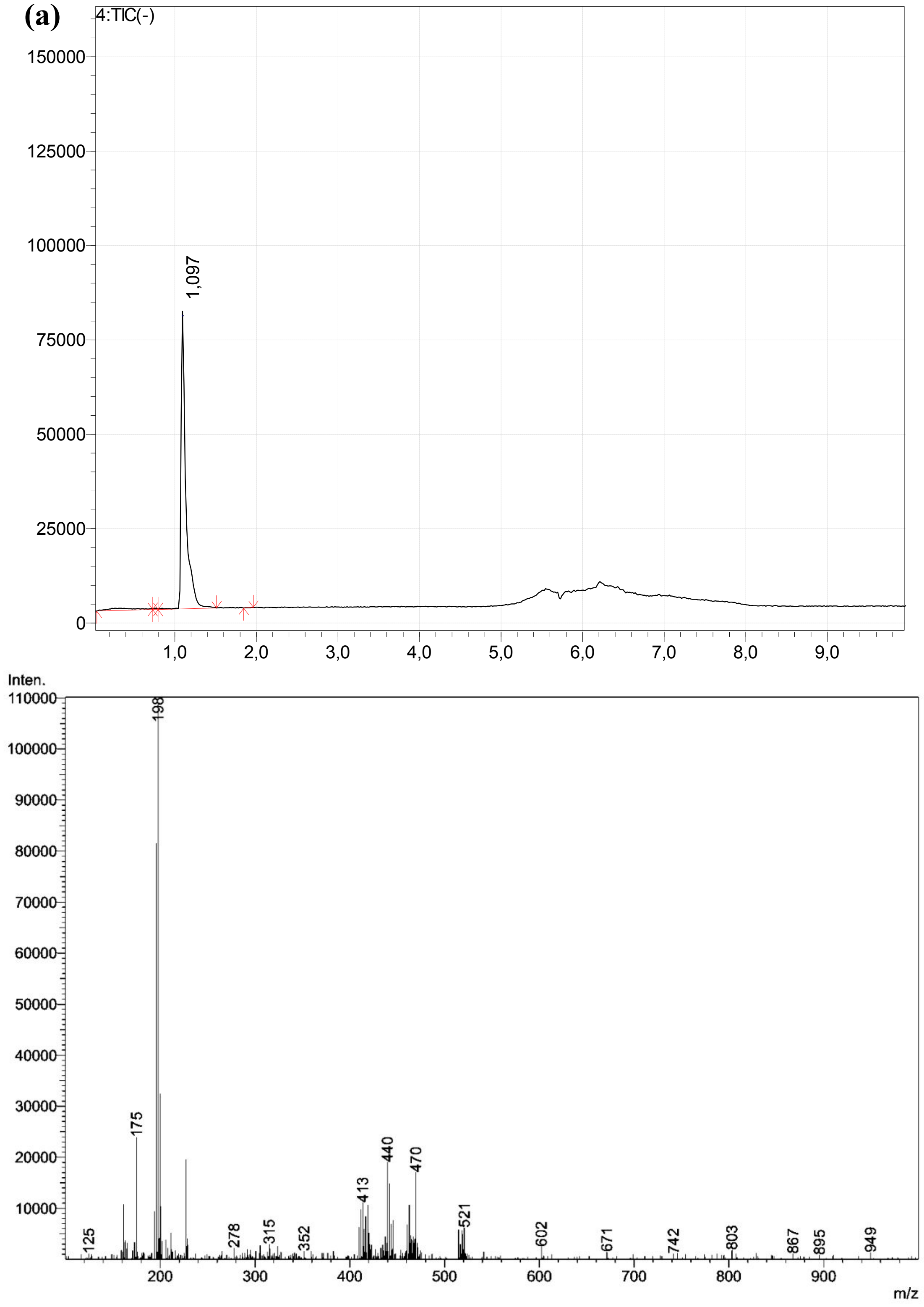

(b)

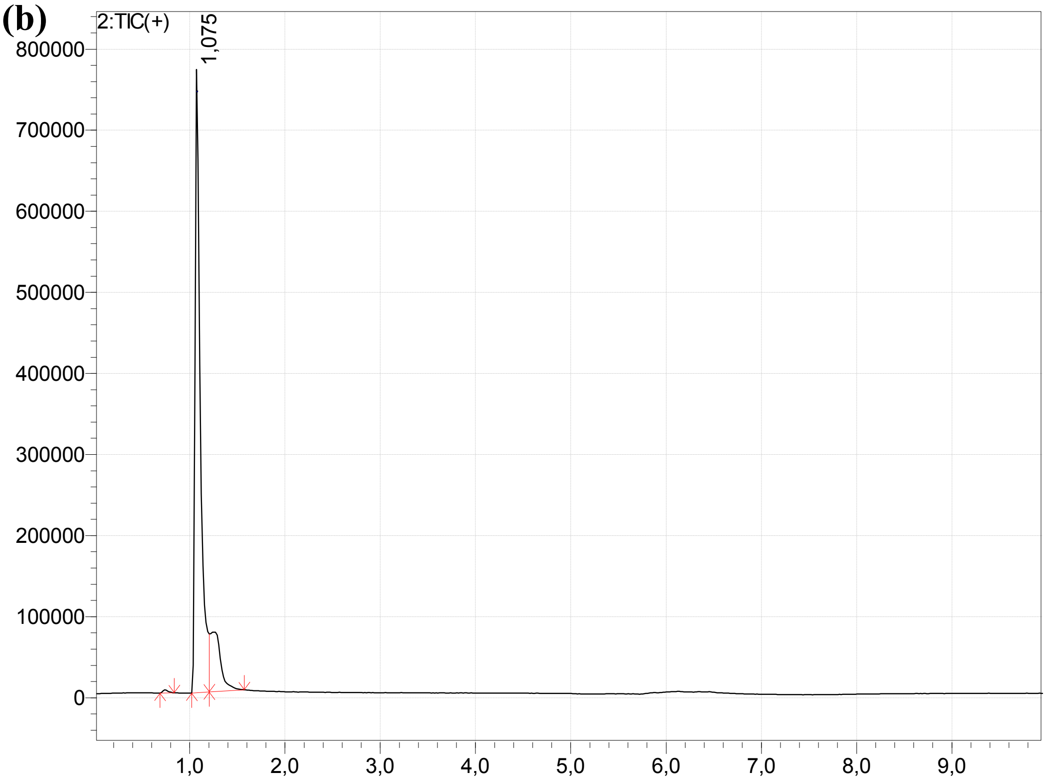
2:TIC(+)
1,075
800000
700000
600000
500000
400000
300000
200000
100000
0
1,0
2,0
3,0
4,0
5,0
6,0
7,0
8,0
9,0

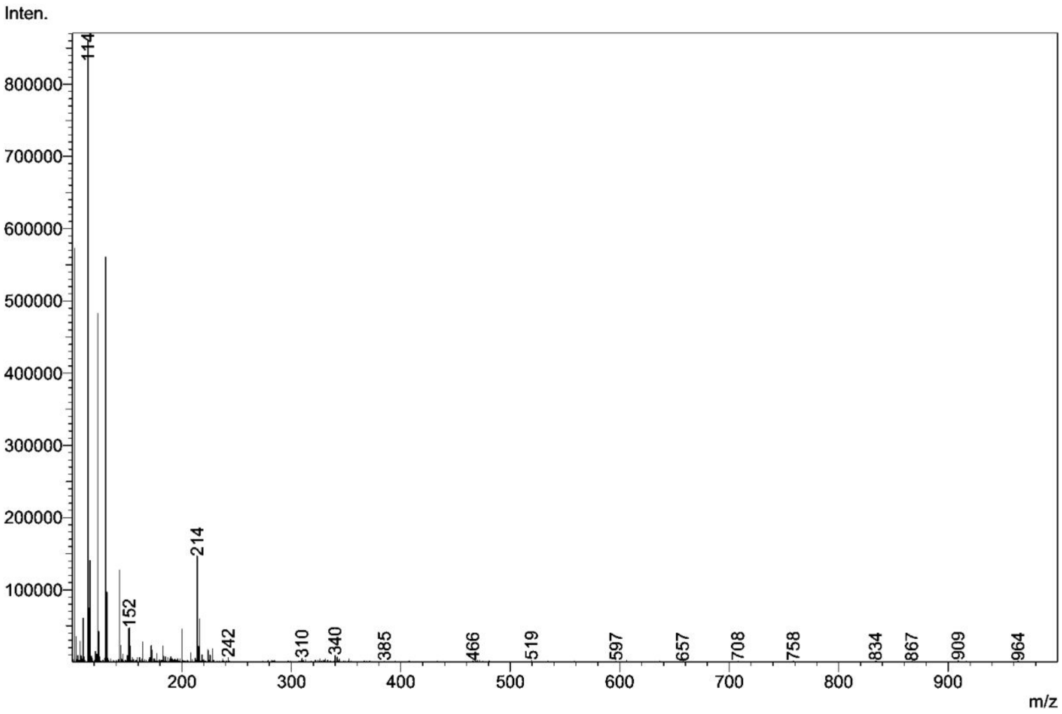
Inten.
114
800000
700000
600000
500000
400000
300000
200000
100000
214
152
242
310
340
385
466
519
597
657
708
758
834
867
909
964
200
300
400
500
600
700
800
900
m/z

Figure S3. Trials for higher-order derivatives of ASC and NIC: **(a)** second-order and **(b)** third-order.

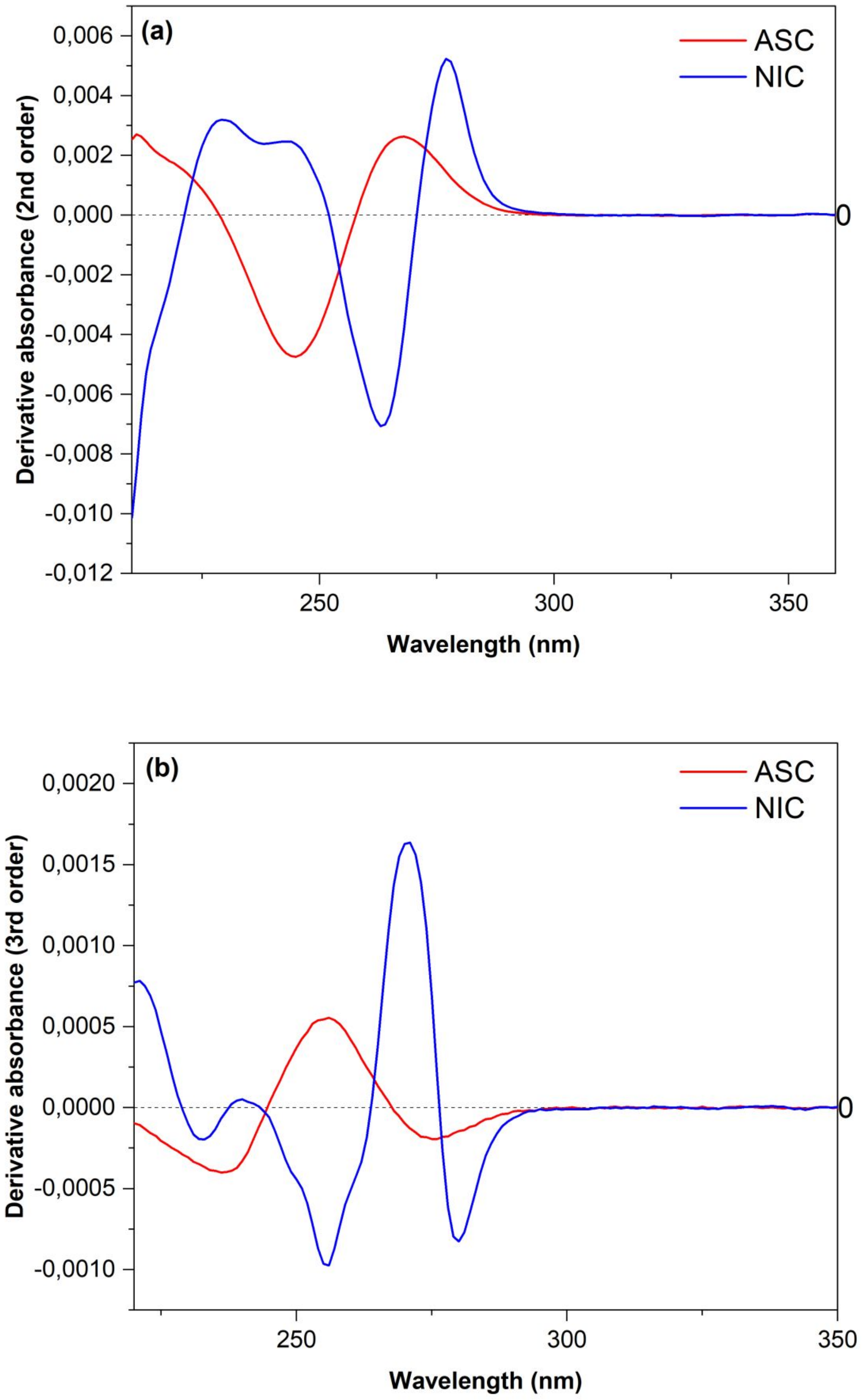

APPENDIX A – Cocrystal purity calculations (According to Reference n. 32)

Since the bulk powder obtained by GAS is formed by cocrystals with some excess ASC homocrystals (ASC_{hc}), all NIC present (NIC_{total}) is the same from the cocrystals (NIC_{cc}), while not all ASC (ASC_{total}) in the samples is from cocrystals (ASC_{cc}), resulting in Equations (1) and (2):

$$m(NIC)_{total} = m(NIC)_{cc} \ (1)$$

$$m(ASC)_{total} = m(ASC)_{cc} + m(ASC)_{hc} \ (2)$$

From the global mass balance on the collected mass (m_{col}) for an individual GAS experiment:

$$m_{col} = \ m_{cc} + m(ASC)_{hc} \ (3)$$

The superscripts 'cc' and 'hc' refer to the masses of cocrystals and homocrystals, respectively. Once the cocrystal is formed at a molar ratio of 1.0:

$$\frac{n(ASC)_{cc}}{n(NIC)_{cc}} = 1 \ (4)$$

Applying the relationships (5) and (6) in (4), we obtain (7):

$$n(ASC)_{cc} = \frac{m(ASC)_{cc}}{MM_{ASC}} \ (5) \qquad n(NIC)_{cc} = \frac{m(NIC)_{cc}}{MM_{NIC}} \ (6) \qquad \frac{m(ASC)_{cc}}{m(NIC)_{cc}} = 1 \times \frac{MM_{ASC}}{MM_{NIC}} \ (7)$$

From the mass balance Eq. (3), by applying Eqs. (1), (2), and (7) we obtain (8).

$$m_{cc} = m_{col} - m(ASC)_{hc}$$

$$m_{cc} = m_{col} - [m(ASC)_{total} - m(ASC)_{cc}]$$

$$m_{cc} = m_{col} - \left[m(ASC)_{total} - m(NIC)_{cc} \times 1 \times \frac{MM_{ASC}}{MM_{NIC}}\right]$$

$$m_{cc} = m_{col} - m(ASC)_{total} + m(NIC)_{total} \times \frac{MM_{ASC}}{MM_{NIC}} \quad (8)$$

Diving (8) in terms of the collected mass, the ratio between masses of cocrystal (m_{cc}) and m_{col} represents the purity of the sample, Eq. 9.

$$Purity\ (wt\ \%) = \frac{m_{cc}}{m_{collected}} \times 100 = \left[1 - \frac{m(ASC)_{total}}{m_{collected}} + \frac{m(NIC)_{total}}{m_{collected}} \times \frac{MM_{ASC}}{MM_{NIC}}\right] \times 100 \quad (9)$$

The collected mass and the total masses of ASC and NIC in samples determined by HPLC/FODS methods are known. The molar masses used for ASC and NIC were 176.12 $g{\cdot}mol^{-1}$ and NIC 122.12 $g{\cdot}mol^{-1}$, respectively.
